# Social Class Priming Effect on Prosociality: Evidence from Explicit and Implicit Measures

**DOI:** 10.3390/ijerph19073984

**Published:** 2022-03-27

**Authors:** Shan Zhang, Xinlei Zang, Sainan Zhang, Feng Zhang

**Affiliations:** Institute of Psychology and Behavior, Henan University, Kaifeng 475004, China; xxxzhangs@126.com (S.Z.); terry_zang@163.com (X.Z.); 13166397176@163.com (S.Z.)

**Keywords:** social class priming, prosocial behavior, donation, single category implicit association

## Abstract

Although abundant research has explored the relationship between social class and prosociality, it remains controversial. This study aimed to investigate the effect of social class priming on prosociality among college students. Experiment 1 was an explicit experiment in which we employed the MacArthur scale to prime participants’ social class and then used a donation task. The results showed that students in a low social class priming group had more donation behaviors compared to ones in a high social class priming group. Experiment 2 was an implicit experiment in which we used a single category implicit association test (SC-IAT) to investigate the relationship between the self-concepts of different social classes and prosociality after priming participants’ social class. The results indicated that students in a low social class priming condition had a stronger connection between self-concepts and prosocial inclinations than ones in a high social class priming condition. Thus, our study demonstrated that students primed with low social class were more prosocial than those primed with high social class, and supported the empathy-altruism theory of prosocial behavior. These findings are of great practical significance to promote prosocial behavior of individuals of different social classes.

## 1. Introduction

Prosocial behavior was defined as voluntary behavior intended to benefit another [1]. Prosocial behavior had important implications for both the individual and society. For many adolescents and adults, prosocial behavior improved health and well-being [2], and for society, prosocial behavior could increase the wealth of societies [3]. In addition, prosocial behavior supported the functioning of a great range of relationships in society [4].

Several theories on the origin of prosocial behavior have been proposed. Kin selection theory [5] holds that humans are more likely to help relatives than non-relatives because this behavior is beneficial to their gene development and survivals. According to social norm theory [6], humans will learn social norms in the socialization process so that they are recognized by society. Meanwhile, prosocial behavior is of benefit to society, and it internalizes and regulates people’s code of conduct. Social norms can lead people to act prosocially if necessary. The reciprocity theory [7] suggests that people are willing to help those who previously supported them. In addition, when people experience empathy, they often seek to support other people’s well-being [8]. Empathy-altruism theory [9] points out that as a factor susceptible to social categorization, empathy tends to have positive effects on in-group prosocial behaviors without affecting intergroup [10]. This means that when helpers have the same social class as recipients, they are more likely to show prosocial behavior due to empathy.

Prosocial behavior is closely connected to social class. For most modern humans, social class is a profound dimension of our social life [11]. Social class refers to a category rooted in an individual’s wealth, education, and occupational prestige [12]. Additionally, a large number of studies have demonstrated a remarkable effect of social class on one’s daily life, such as thought, feelings, and behavior [13,14,15,16,17,18,19]. 

Some researchers argued that low social class people were more prosocial toward others than those with high social class [20]. Previous studies [21] found that compared with high social class students, low social class students exhibited more prosocial behaviors. They also found that the students from low-income families were more likely to help each other when they experienced difficulties. Previous findings [22] emphasized that low social class individuals were more likely to engage in others’ beneficial prosocial behaviors, whereas high social class individuals were more likely to engage in self-beneficial behaviors. According to the social cognitive theory of social class [11], prosocial behavior was an adaptive strategy among people with lower social class. Lower class individuals’ wealth and education were uncertain, and they would experience more risk factors that could not be controlled. Thus, they showed a lower autonomy and tended to cooperate with others, which led to more prosocial behaviors [11,15,22]. 

However, there were opposite opinions of the relationship between social class and prosocial behavior. Researchers pointed out that high social class individuals were more likely to engage in prosocial behaviors than low social class ones [23,24]. Eight studies revealed that [23] higher class people were more likely to make charitable donations and do volunteer work, and be more trusting. The latest field experiment showed high social class people behaved more prosocially than low social class people [25]. Furthermore, researchers found that people in the upper social class were more prosocial in public than in private, whereas the reverse was true for people with lower social class [26]. In sum, those studies have pointed toward a positive effect of social class on prosocial behavior.

Moreover, numerous studies have found that there was less reproducibility in psychology, and it faced a replication crisis. According to a research article summary [27], there was scope to improve reproducibility in the field of psychology. The replications increased confirmatory and deterministic evidence. Low replicability would decrease public trust in psychology [28], which also affected the development of science [29,30]. However, the repeated frequency of many findings was below expectations [31]. A lot of previous research did not replicate efficiently in the new research. Such a way may easily lead to erroneous conclusions [31]. Thus, we expected that most of the previous statistically significant results would be replicated in new data. 

Given that previous research has shown controversial results, and in order to gain a broader understanding of the relation between social class and prosociality, the current study tried to investigate the effects of social class priming on prosocial behavior with two separate approaches. Against this background, Experiment 1 used an explicit task to seek to validate the reliability of previous study results that lower social class individuals were more prosocial. Previous studies used different experimentation methods to measure prosocial behavior. The dictator game, donation task, and trust game were the most representative methods [23,32,33,34]. As in previous studies [35,36], we chose a donation task to measure the explicit prosocial behavior in Experiment 1. We used an implicit measure (a single category implicit association test, SC-IAT) to retest the effect of social class priming on prosociality in Experiment 2. Based on the results of previous studies [22], the hypotheses we proposed were as follows: (1) The amount donated by individuals primed with low social class was significantly more than those primed with high social class; (2) The association between self-concepts and prosocial inclinations of individuals primed with low social class was significantly stronger than those primed with high social class. 

## 2. Experiment 1

The aim of Experiment 1 was to explore whether there was a remarkable effect of social class priming on prosocial behavior. We defined the average donation amount in Experiment 1 as an explicit indicator of prosocial behavior. 

### 2.1. Material and Method

#### 2.1.1. Participants

The sample included 46 students (M _age_ = 20.54 years old, SD _age_ = 1.19; 22 females) who were voluntarily recruited from a university in Henan province in China via the Internet. All participants were right-handed with normal or corrected vision and without a neurological or psychiatric history. All participants provided informed consent before this experiment. 

#### 2.1.2. Procedures and Materials

Before the experiment started, all participants completed an online survey with an objective social class questionnaire. First, upon arrival to the lab, the participants were randomly assigned to one of two groups (experimental priming: high social class vs. low social class), and their subjective social class was manipulated. Next, they completed the MacArthur Scale [37] and reported their perceived social classes from a 10-rung ladder in their current states. Then, they fulfilled the donation task [38]. In the end, the participants received CNY 30 as their rewards.

**Figure 1:**
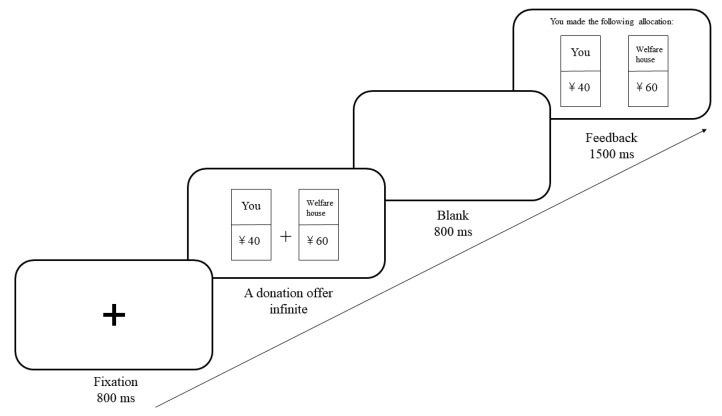
Flow diagram of a donation task trial.

1. Objective social class questionnaire. We used participants’ parents’ education level, occupational prestige, and family income to measure objective social class [20]. Participants rated their parents’ educational level based on five categories: (1) Primary school or less education; (2) Junior middle school; (3) High school graduation; (4) College education; or (5) Graduate-level education. The occupation was classified into five categories: (1) Agricultural laborer, unskilled worker, or unemployed people; (2) Manual worker, self-employed person, or skilled worker; (3) Ordinary manager, or junior professional technician; (4) Middle manager, or intermediate professional technician; or (5) Senior manager, or senior professional technician. Participants rated their monthly family income (CNY) based on 12 categories: (1) Less than 2000; (2) 2000–3000; (3) 3000–4000; (4) 4000–5000; (5) 5000–6000; (6) 6000–7000; (7) 7000–8000; (8) 8000–9000; (9) 9000–10,000; (10) 10,000–11,000; (11) 11,000–12,000; or (12) More than 12,000. According to previous research [39], we calculated a composite measure of the total social class scores by summing the standard *Z*-scores of education level, occupational prestige, and monthly family income.
2. Subjective social class manipulation. We used the MacArthur Scale [36] to manipulate participants’ social class priming. The participants saw a picture of a ladder with ten rungs and were asked to imagine that the ladder represented different social classes in China: Lower (higher) position of the ladder referred to lower (higher) social class. People at the bottom (top) were worse (best) off in terms of income, education, work, and living conditions. In the high social class priming group, participants were requested to compare themselves with the lowest class and then thought of talking to someone at the bottom. In the low social class priming group, participants were requested to compare themselves with the highest class and then thought of talking to someone at the top.
3. Donation task. We measured participants’ prosocial level with the donation task (see Figure 1). At first, a fixation was shown in the middle of the screen for 800 ms. Next, a donation offer was presented, and participants were asked to decide whether to donate. There were 20 donation offer ways (you—welfare house): CNY 0–100, 5–95, 10–90, 15–85, 20–80, 25–75, 30–70, 35–65, 40–60, 45–55, 50–50, 55–45, 60–40, 65–35, 70–30, 75–25, 80–20, 85–15, 90–10, or 95–5. Participants pressed “F” key if they agreed to this donate offer way. Participants pressed “J” key if they did not agree, and then all the money would be kept by them. Afterwards, a blank screen was displayed for 800 ms, and then feedback was presented for 1500 ms. This task consisted of two blocks, and each block had 80 trials. Approximately 5–10 min of eye-closed resting after a block. The type of the two keys was counterbalanced among participants.

### 2.2. Results and Discussion 

First, we examined the baseline data of objective social class, and the results showed no significant difference between high social class priming group and low social class priming group in the objective social class (M ± SD _low_ = 0.319 ± 4.41, M ± SD _high_ = −0.256 ± 4.42; *t* = 0.442, *p* = 0.661) before the start of manipulation. Then, an independent samples t-test was used as priming manipulation checks. As expected, the results showed that participants in the high social class priming group perceived higher social class than those in the low social class priming group (M _high_ = 5.88, SD _high_ = 1.25; M; _low_ = 4.05, SD _low_ = 1.03; *t* = 5.421, *p* < 0.001). It proved that our subjective social class manipulation was effective.

Further analyses revealed a significant group difference in donation amount (*t* = 2.23, *p* = 0.031). The amount of donation was significantly greater in the low social class priming group than in the high social class priming group (M _low_ = 31.99, SD _low_ = 12.80; M _high_ = 23.34, SD _high_ = 13.45). No significant difference was found between males and females (*t* = −0.133, *p* = 0.895). Overall, these results supported our first hypothesis.

Although Experiment 1 demonstrated that people primed with lower social class had more prosocial behaviors, the donation task was explicit and it was susceptible to participants’ motivation of self-presentation. The result of a single category implicit association test (SC-IAT) might be a good prediction of participants’ spontaneous behavior [40]. Thus, a SC-IAT was conducted to validate whether priming social class affected implicit prosocial attitudes in Experiment 2.

## 3. Experiment 2

### 3.1. Material and Method

#### 3.1.1. Participants

The sample included 50 students (M _age_ = 20.74 years old, SD _age_ = 1.48; 23 females) recruited voluntarily from a university in Henan province in China via the Internet. All participants were right-handed with normal or corrected vision and without a neurological or psychiatric history. All participants provided informed consent before the experiment.

#### 3.1.2. Procedures and Materials

Before the experiment started, all participants completed an online survey with an objective social class questionnaire. Upon arrival to the lab, participants were randomly assigned into two groups (experimental priming: high social class vs. low social class), and the same subjective social class priming manipulation was conducted as Experiment 1. Then, participants were required to complete the SC-IAT. Finally, the participants were paid CNY 30 as their rewards. 

In SC-IAT, the experiment was programmed using E-prime 2.0 to test participants’ implicit associations among five self words, five prosocial words, and five non-prosocial words. The self words were as follows: “我” (“me”), “我的” (“mine”), “我们” (“us”), “自己” (“myself”), and “咱们” (“ourselves”). The prosocial words were as follows: “捐款” (“donation”), “捐资” (“endowment”), “捐钱” (“contribution”), “资助” (“grant”), and “救济” (“relieve”). The non-prosocial words were as follows: “索要” (“ask for”), “索取” (“demand”), “索拿” (“claim”), “抢占” (“extort”), and “讨要” (“beg”).

**Figure 2:**
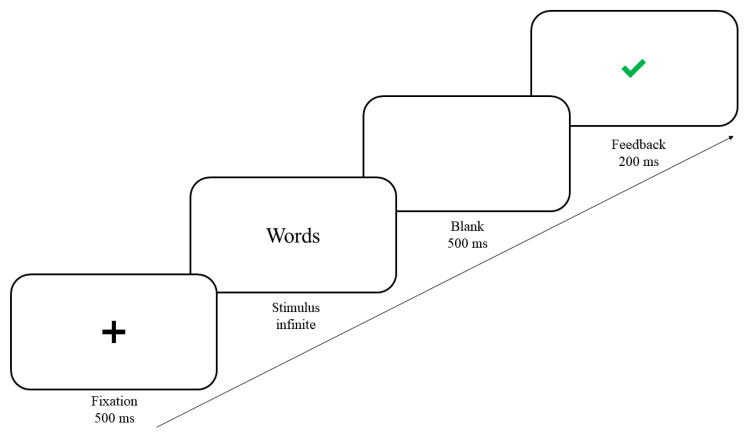
Flow chart of a SC-IAT trial.

SC-IAT consisted of two tasks (a congruent task and an incongruent task), each of which consisted of 20 practice trials followed by 80 test trials. In a typical trial (see Figure 2), a fixation cross appeared for 500 ms at the center of the screen. A word was then presented until participants responded, followed by a blank screen for 500 ms. In the congruent task, participants were asked to press the “F” key with their left index finger when they saw a self word or a prosocial word, and press the “J” key with their right index finger when they saw a non-prosocial word. In the incongruent task, participants were asked to press the left-hand response “F” key if the stimulus was a prosocial word, and press the right-hand response “J” key if the stimulus was a self word or a non-prosocial word. In order to prevent response bias, self words, prosocial words, and non-prosocial words were presented in a 1:1:2 ratio in the congruent condition, and there was a 1:2:1 ratio (self words, prosocial words, and non-prosocial words) in the incongruent condition, such that the corrected responses were split equally between the F and J key. Finally, there was feedback for 200 ms, in which a green “√” was presented when the participants’ response was correct; Otherwise, a red “×” was presented [41]. The order of the congruent task and the incongruent task was counterbalanced between participants, and the trial order was randomized.

### 3.2. Results and Discussion

First, we examined the baseline data of objective social class, and the results showed no significant difference between high social class priming group and low social class priming group in the objective social class (M ± SD _low_ = 0.27 ± 4.416, M ± SD _high_ = 0.81 ± 4.144; *t* = −0.446, *p* = 0.658) before the start of priming manipulation. Then, manipulation check analyses indicated that participants in the high social class priming group perceived higher social class (M _high_ = 5.96, SD _high_ = 1.24; M _low_ = 3.96, SD _low_ = 1.10; *t* = 6.03, *p* < 0.001). The result showed that the subjective social class priming manipulation was effective.

**Figure 3:**
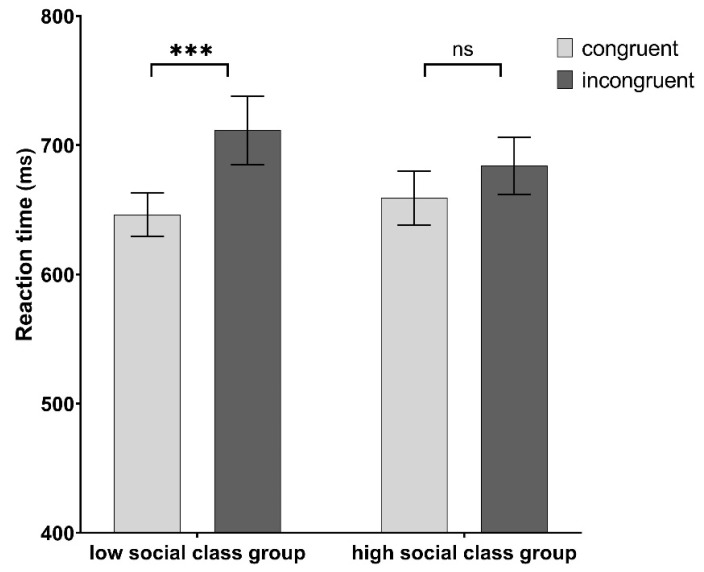
Mean (and standard error) of reaction time of different groups in congruent task and incongruent task (*** *p* < 0.001).

We further conducted a paired samples t-test (see Figure 3). Results revealed that for low social class priming group, the reaction time of the congruent task was significantly faster than the incongruent task (M ± SD _congruent_ = 646.35 ± 84.23, M ± SD _incongruent_ = 711.50 ± 131.81; *t* = −3.873, *p* < 0.001). It suggested that their self-concepts showed a close association with prosocial attitudes at the level of implicit processing. However, for high social class priming group there was no significant difference in the reaction time between the two tasks (M ± SD _congruent_ = 659.15 ± 104.13, M ± SD _incongruent_ = 684.11 ± 110.17; *t* = −1.929, *p* = 0.066). These results confirmed our second hypothesis.

## 4. General Discussion

In previous studies on social class effect on prosociality, implicit and explicit measures were always isolated, and few studies combined the two methods [34,42,43]. We conducted an implicit experiment and an explicit experiment to test whether lower social class tended to be more prosocial. In Experiment 1, we found that the donation in low social class priming group was significantly higher than in high social class priming group, which was consistent with previous research [15,20,21,22]. Experiment 2 employed an implicit task of SC-IAT, which was a more accurate measure of implicit attitude [41] to explore the degree to which prosocial behaviors were integrated into the participants’ self-concepts. We found that the low social class priming group had stronger associations between self-concepts and prosocial inclinations than the high social class priming group. In a word, our work further verified and extended the existing studies [20,22].

The results of this study supported the empathy-altruism theory. This theory [9] suggested that empathic emotions (e.g., compassion, sympathy) aroused altruistic motivation. The researchers [44] suggested that empathy attuned people to others’ needs. This was probably because empathy induced an altruistic desire to increase the other’s benefit. A feeling of empathy aroused altruistic motivation to improve the welfare of the people in need [45]. Furthermore, low social class individuals experienced more compassion than high social class individuals [46]. People with low class had stronger empathy than those with high class when they saw negative, painful pictures [46,47]. Empathy had a positive effect when it came to intragroup helping [10], and people were more likely to help others in their own group [48]. People were likely to help others when they had a sense of self-other overlap. Therefore, low social class individuals were likely to be prosocial when they were confronted with people who were in adversity. In our study, the welfare house represented the lower social class in a way. So, people in low social class priming group had more prosocial behaviors and implicit prosocial attitudes.

Our study results indicated that the students primed with high social class had no significant implicit prosocial attitudes. According to the social cognitive theory of social class [11], substantial resources and high rank of higher social class individuals offered a context that enhanced the freedom and induced the solipsist social cognitive pattern. In this sense, high social class individuals focused on their own internal goals, emotions, and motivations rather than external environmental forces and others’ emotions [11]. Therefore, students in high social class priming group showed less implicit prosocial attitudes and prosocial behaviors than ones in low social class priming group.

The present study was not without limitations. Firstly, previous studies revealed that context, resources, and social class stereotyping were linked to prosocial behavior [26,49,50]. Future research could explore the interaction effects between these potential variables and social class to validate the conclusion of our present study. Secondly, prosocial behavior was a broad concept covering many manifestations (e.g., cooperation, trust, and help) [45]. We hope that future studies will be conducted through different indicators to deepen our current research. Thirdly, it is difficult to rule out the influence of social desirability or other motives for the donation task in Experiment 1. Future research should improve this task paradigm to verify our results. Lastly, the generalizability of our results should be cautious because the samples of participants were college students, future research could collect data from non-student samples and expand the sample sizes of participants appropriately.

## 5. Conclusions

Compared to those in high social class priming group, students in low social class priming group were more likely to donate, and their self-concepts were more closely associated with prosocial attitudes in implicit processing. Therefore, students primed with low social class were more prosocial than ones primed with high social class. Our results provided supporting evidence for empathy-altruism theory of prosocial behavior.

## Data Availability

The data presented in this study are available on request from the corresponding author.
